# Microvascular disease and severe COVID-19 outcomes in UKBiobank participants with diabetes

**DOI:** 10.1007/s00592-024-02420-z

**Published:** 2024-11-21

**Authors:** Claire Tochel, Justin Engelmann, Ylenia Giarratano, Baljean Dhillon, Roly Megaw, Miguel O. Bernabeu

**Affiliations:** 1 Centre for Medical Informatics, Usher Institute, University of Edinburgh, Edinburgh, EH16 4UX UK; 2 Centre for Clinical Brain Sciences, Chancellor’s Building, University of Edinburgh, Edinburgh, EH16 4SB UK; 3 MRC Human Genetics Unit, University of Edinburgh, Edinburgh, EH4 2XU UK; 4 Princess Alexandra Eye Pavilion, NHS Lothian, Chalmers St, Edinburgh, EH3 9HA UK; 5 The Bayes Centre, University of Edinburgh, Edinburgh, EH8 9BT UK

**Keywords:** Coronavirus, Microvascular, Diabetes, Retinopathy, UKBiobank, Retrospective cohort

## Abstract

**Aims:**

Early in the COVID-19 pandemic, evidence emerged suggesting that people with diabetic retinopathy (DR) or other microvascular diseases had greater risk of severe short-term outcomes. This study evaluated longer-term outcomes, providing more generalisable evidence.

**Methods:**

We identified a cohort of UKBiobank participants with diabetes and retrieved their diagnostic codes for a variety of microvascular diseases, complications of diabetes and systemic comorbidities. We investigated relationships between diagnoses and the study outcome: admission to Critical Care or death from COVID-19, taking age, sex and diabetes duration into account. We tested relationships, adding baseline covariates and weighting diagnostic codes according to their recency prior to COVID-19 diagnosis.

**Results:**

In univariate analyses, DR (OR: 1·519, *p* = 0·016) and microvascular disease (OR: 2·001, *p* = 0·000) were associated with greater risk of the outcome. In multivariate analyses, as expected, respiratory disease was most strongly associated with the study outcome, microvascular disease second. Adjusting analyses by number of admissions (general health proxy) and weighted diagnostic coding (comorbidity severity at COVID-19 diagnosis indicator), did not improve predictive power of the model.

**Conclusions:**

The presence of microvascular disease in routinely-collected healthcare data predicts risk of COVID-19 severe outcomes, independently of general health, in a cohort of people with diabetes.

**Supplementary Information:**

The online version contains supplementary material available at 10.1007/s00592-024-02420-z.

## Introduction

During the COVID-19 pandemic, evidence emerged to suggest that Diabetic Retinopathy (DR) and other microvascular diseases were associated with severe outcomes among people with diabetes [1–6]. COVID-19 is known to manifest as thromboembolic microvascular disease, prompting proposed pathomechanisms which included direct or indirect activation of endothelial exocytosis, thereby mediating vascular inflammation [7].

Early COVID-19 research was commonly undertaken in acute hospital settings where large numbers of patients were treated by clinicians observing and learning about the pathway of the disease under intense and unprecedented pressure. They, by necessity, often reported on short-term outcomes such as fatality at seven [1] or 28 [6] days, admission to Critical Care Unit (CCU) [4, 5, 8] or intubation [1, 2]. DR and microvascular disease were identified as two of many risk factors associated with each study outcome, and their predictive power understandably varied by outcome definition, including statistically significant odds ratios (ORs) for the association between DR and study outcomes where the outcome was death or CCU admission (OR 1·67) [4], or death at day seven (OR 2·05) [1] or intubation (OR 5·81) [9]. Further variation in reported associations depended on which covariates were incorporated into analysis, and the inclusion criteria for diabetes (most studies included primarily or entirely people with type 2 DM) and DR (any [5, 8, 9], severe [1], referable / non-referable [4]). A systematic review summarised all available evidence from the 1st (March to July 2020) and 2nd (September to December 2020) waves of the pandemic, highlighting the need for the analysis of longer-term outcomes of COVID-19 for people with diabetes with more generalisable results [10].

This analysis builds on these early clinical studies using longitudinal, linked healthcare data from UKBiobank (UKB) which provides rich data on a geographically dispersed and relatively representative UK population cohort. Further, these data allow us to evaluate severe outcomes of COVID-19 beyond the limits of a hospital episode, which increases the real-world relevance and broadens the generalisability of the findings. We report on such outcomes (following STROBE guidelines) [11] among UKB participants before the impact of wide vaccination roll out, and have attempted to control for.

We describe the phenotypic characteristics of UKB participants with diabetes who contracted COVID-19 and explore the relative association between DR and other microvascular diseases on risk of severe outcomes by answering the following research questions.

1. Is DR or microvascular disease associated with severe outcomes of COVID-19?
2. How does this compare to other comorbidities: other specific microvascular diseases, other complications of diabetes and systemic comorbidities?
3. How does this ranking of association change if we include hospital admissions as a proxy for general patient health?
4. Which group of comorbidities is the most informative about severe outcomes of COVID-19?

## Methods

Data were downloaded from UKB in May 2021 [12]. UKB is a large biomedical database which contains detailed health information on around 500k participants from the UK. Participants were recruited in 2006-10 at which time they were aged 40 or over. Potential participants were randomly invited, with stratification, from the eligible population to ensure that they were representative of the UK in several ways (e.g. sex, ethnicity, country) but participation was voluntary and therefore self-selecting, resulting in a group which is generally healthier and less deprived than the UK population. This study included data collected from participants at recruitment and several linked datasets: hospital in-patient, critical care, primary care, death and COVID-19 test data.

Our study period was set by the first confirmed UK COVID-19 case (31st January 2020). We included all participants with diabetes, alive three weeks before that date (10th January 2020). We identified all COVID-19 cases until 23rd February 2021, with outcomes up to 28 days later.

### Diabetes cohort identification

We identified UKB participants with diabetes using a variety of source data. This included self-reported diagnoses at recruitment, relevant diagnostic codes (diabetes, DR) and in hospital in-patient, primary care or death records [13]. Further cases were identified in primary care using Hba1c measurements and a set of inclusion and exclusion codes [14]. Primary care data is not available for all UKB participants, so some potentially relevant individuals were not included in this cohort.

Demographic information on cohort members (age, sex, BMI, deprivation, ethnic group) were taken from self-reported information at recruitment.

### Primary outcome definition

The study’s primary outcome was defined as either admission to CCU or death associated with COVID-19. These states were identified in the data through death or CCU admission record which included a COVID-19 ICD10 code (U071, U072); or a death or CCU admission record within 28 days of a positive COVID-19 test.

For individuals with multiple positive COVID-19 tests, the time between the positive test and any record of CCU admission or death was sought. Where multiple instances met the timing criterion (i.e. under 28 days) the date of the first positive test was used.

### DR and comorbidities definition

For each person with diabetes, we looked for ICD10 codes for DR and a range of other comorbidities in their hospital in-patient data. These comorbidities were identified as relevant from several published studies which investigated predictors of severe COVID-19 outcomes [1, 3–5, 15]. Within the category of microvascular disease we included DR, nephropathy, neuropathy, foot ulcer and chronic kidney disease. Other complications of diabetes comprised hypoglycaemia, diabetic ketoacidosis, peripheral arterial disease, heart disease and stroke. Finally, systemic comorbidities comprised nervous or respiratory system disease, chronic liver disease and immunodeficiency.

ICD10 code lists for many of the above conditions were published by McGurnaghan et al. and adapted for these data [4]. Others were identified using the WHO browser and by pattern matching relevant terms within a list downloaded from NHS Digital [16, 17]. New code lists were reviewed by a clinician for relevance.

Hospital in-patient data were available for all UKB participants back to 1998. The earliest code per person was compared to the study end date or, where relevant, their “COVID-19 date” i.e. the first date on which it was identified that they had COVID-19 by either definition above. If the comorbidity occurred for the first time after this date, that individual was not flagged as having the comorbidity.

### Analysis of association with primary outcome

For each research question, we tested for an association with the primary outcome, initially taking age, sex and diabetes duration into account (simple baseline). Subsequent analyses involved an extended baseline: ethnicity, deprivation index, BMI, smoking status, systolic blood pressure and admissions since 1998. Logarithmic transformation normalised the very positively skewed range in number of admissions.

Our candidate variables were the comorbidities. We considered baseline variables in combination with each candidate variable individually. From all models, we picked the one that gave the best criterion and considered it in combination with each remaining candidate variable individually. We repeated this process until our criterion stopped improving or we ran out of variables, but for clarity, the representative plots (e.g. Figure 2) include twelve iterations only. We used the Bayesian Information Criterion (BIC) as it is selective and yields a parsimonious model; log-likelihood penalised for the number of fitted parameters (conceptually the same as an adjusted R^2^). We plotted Akaike Information Criterion (AIC) for reference.

Missing data were imputed with the median value for numerical variables and the mode for categorical variables. Missing rates, with median or mode are provided as supplementary material (SM).

The analytical approach for each research question is outlined below.

1. DR & microvascular disease.

We fitted a logistic regression for DR and microvascular disease individually using the primary outcome as the dependent variable, controlling for age, sex, and diabetes duration, and reported the odds ratio and p-value.

2. Other comorbidities.

We compared model fit (log likelihood) through logistic regression for each comorbidity separately (static comparison) plus age, sex, diabetes duration (as well as this baseline without any comorbidity) and assessed how well it explained the data. We looked at AIC/BIC which had the same penalty for each comorbidity model, telling us which comorbidities do not substantially improve over the baseline. As adding more parameters guaranteed a better fit, use of AIC/BIC provided a way of gauging whether the improvement was better than random.

3. Hospital admissions.

As above, we conducted a static comparison of models per comorbidity adjusted for age, sex, diabetes duration but used a more complex baseline which included ethnicity, deprivation index, BMI, smoking status, systolic blood pressure and hospital admissions (log transformed).

4. Most informative comorbidities.

To assess the relative predictive power of each comorbidity, we conducted a stepwise selection of each (where predictive variables are chosen by an automated procedure to maximise performance) and reported the OR/p-value for the selected model.

## Results

Among 473,737 UKB participants alive at the start of the study period, 49,831 (10·5%) were identified as having evidence of diabetes either by self-report of through linked healthcare records. Just under 2,500 (5·2%) of these tested positive for COVID-19 during the study period. Demographic characteristics are provided as SM (Table 4). This showed that people with COVID-19 and the primary outcome were older (74 vs. 69, *p* < 0·0001) than those with COVID-19 without the primary outcome, straddling the age of those who didn’t contract COVID-19 at all (71 years). More COVID-19 positive individuals were male (69 vs. 58%, *p* < 0·0004) compared to 56% of those without COVID-19. Black and minority ethnic groups were over represented in the two COVID-19 groups, e.g. 11 and 7% of those with and without the primary outcome, respectively, were Asian compared to 6% who didn’t contract COVID-19. This difference between the two groups with COVID-19 was statistically significant (*p* = 0·0018). A higher percentage of people from the two COVID-19 groups were from deprived areas (15 and 11% compared to 9% in the group without COVID-19). Among these two sub-groups (high deprivation and Asian ethnicity), a lower percentage of people with COVID-19 experienced the outcome. Notably, the group who never tested positive for COVID-19 also contains more people from deprived areas than the UKB cohort as a whole, reflecting diabetes as a study inclusion criteria and its strong association with deprivation [18]. Being a current or former smoker was more common in people who experienced the primary outcome compared to the other two groups (62 vs. 51%, *p* < 0·0001). Diabetes duration was similar between the two COVID-19 groups (*p* = 0·47).

The number of study cohort members with DR and selected comorbidities, as identified through hospital records, is shown in Table 1 with number of hospital admissions.

**Table 1:** UKB participants with diabetes and one or more ICD10 code for DR and other comorbidity in their hospital inpatient records according to whether they had COVID-19 and the primary outcome

		Never positive COVID-19	1 or more positive COVID-19 test	
			Without 1y outcome	1y outcome
No. of hospital admissions since 1998, median (sd)		7 (22·8)	8 (29·4)	15 (41·5)
Microvascular disease				
DR	No	44,076 (93%)	1826 (92%)	427 (87%)
	Yes	3277 (7%)	163 (8%)	62 (13%)
Nephropathy	No	46,943 (99%)	1958 (98%)	467 (96%)
	Yes	410 (1%)	31 (2%)	22 (4%)
Foot ulcer	No	46,811 (99%)	1946 (98%)	473 (97%)
	Yes	542 (1%)	43 (2%)	16 (3%)
Neuropathy	No	45,594 (96%)	1884 (95%)	441 (90%)
	Yes	1759 (4%)	105 (5%)	48 (10%)
Chronic Kidney Disease	No	42,631 (90%)	1724 (87%)	348 (71%)
	Yes	4722 (10%)	265 (13%)	141 (29%)
Any of the above microvascular diseases	No	39,025 (82%)	1565 (79%)	294 (60%)
	Yes	8328 (18%)	424 (21%)	195 (40%)
Other complications of diabetes				
Hypoglycaemia	No	45,893 (97%)	1898 (95%)	432 (88%)
	Yes	1460 (3%)	91 (5%)	57 (12%)
Diabetic ketoacidosis	No	46,767 (99%)	1964 (99%)	472 (97%)
	Yes	586 (1%)	25 (1%)	17 (3%)
Peripheral arterial disease	No	45,808 (97%)	1907 (96%)	440 (90%)
	Yes	1545 (3%)	82 (4%)	49 (10%)
Heart disease	No	15,535 (33%)	543 (27%)	64 (13%)
	Yes	31,818 (67%)	1446 (73%)	425 (87%)
Stroke	No	45,182 (95%)	1842 (93%)	432 (88%)
	Yes	2171 (5%)	147 (7%)	57 (12%)
Systemic comorbidities				
Nervous system disease	No	42,413 (90%)	1653 (83%)	339 (69%)
	Yes	4940 (10%)	336 (17%)	150 (31%)
Respiratory system disease	No	33,647 (71%)	1264 (64%)	203 (42%)
	Yes	13,706 (29%)	725 (36%)	286 (58%)
Chronic Liver Disease	No	44,188 (93%)	1814 (91%)	401 (82%)
	Yes	3165 (7%)	175 (9%)	88 (18%)
Immunodeficiency	No	46,949 (99%)	1971 (99%)	479 (98%)
	Yes	404 (1%)	18 (1%)	10 (2%)

Median hospital admissions (excluding any after participant’s COVID-19 date or the study end date if they never contracted COVID-19) was higher in people with the study outcome (15 vs. 8) although group means had very large standard deviations. In almost every example, the percentage of people with a comorbidity was highest in the COVID-19 with the primary outcome, then COVID-19 without the outcome and lowest in people who did not contract COVID-19.

The data gave strong indications that those diagnosed with COVID-19 were generally in poorer health than those not diagnosed with COVID-19. This latter group may include untested people with mild or no symptoms who may have stayed home or been unaware that they were positive. Taking covariates and comorbidities into account was therefore a crucial element of the analysis of the relative impact of each comorbidity on the outcome.

First we asked the question if DR and microvascular disease were associated with severe outcomes of COVID-19.

### DR & microvascular disease

Adjusting for age, sex and diabetes duration, both DR (OR: 1·519, *p* = 0·016) and any microvascular disease (OR: 2·001, p = < 0·001) showed a statistically significant positive association for risk of the outcome (Fig. 3 SM). As expected, the OR for people with any of the microvascular diseases (a group which includes DR) was stronger than for DR alone.

When analysed together with microvascular disease, the OR for DR was less than one, indicating that it was a less severe risk than the other microvascular conditions.

**Fig. 1:**
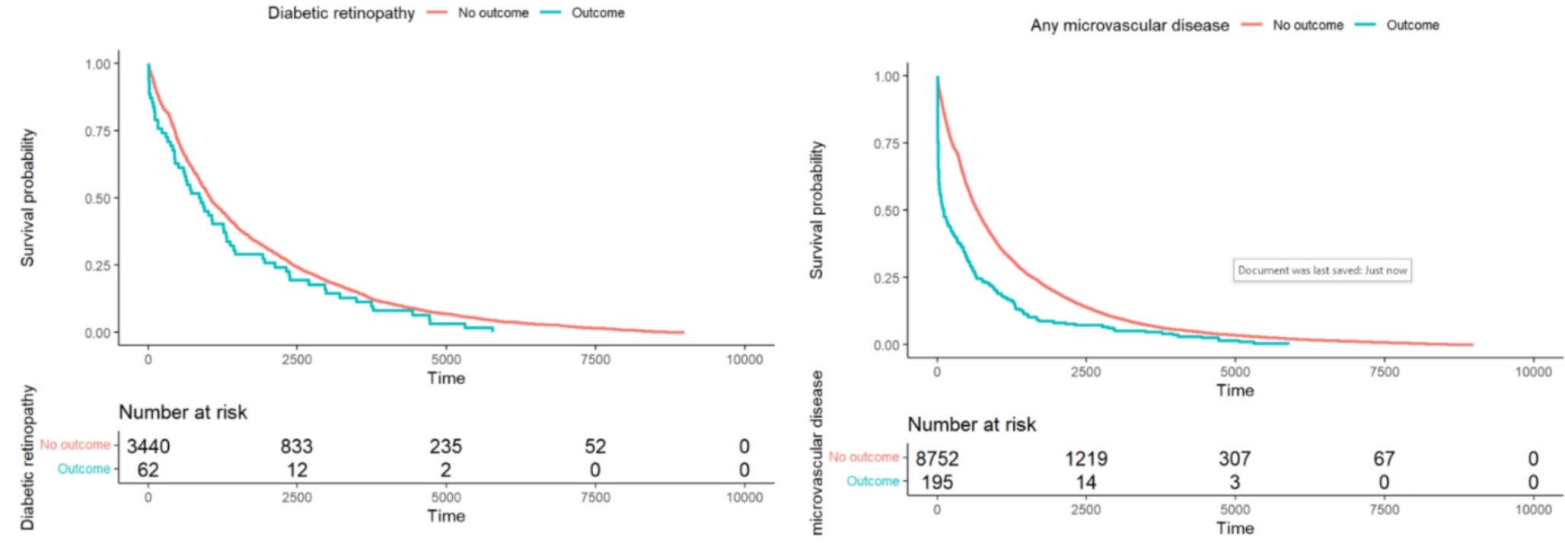
Survival curve to diagnostic code in days for DR (left) or any microvascular disease (right) from end of study (red = outcome not experienced) or first date they experienced study outcome (blue = outcome)

Adjusting for age, sex and diabetes duration, reduction in time to most recent code for DR and any microvascular disease showed a positive association for risk of the outcome (Figs. 1 and 4 SM).

Next, we asked how the associations above compared to other comorbidities.

### Other comorbidities

Table 2 shows the model fit (log likelihood) for static comparison of models per comorbidity adjusted for age, sex and diabetes duration.

**Table 2:** Static comparisons, simple baseline (age, sex, diabetes duration), sorted by BIC with best performing models at the top

	Log-Likelihood	AIC	BIC	*n* (comorbidity)	BIC delta over baseline
Respiratory system disease	-1125	2259	2288	1011	-41
Microvascular disease	-1132	2273	2302	619	-27
Chronic kidney disease	-1135	2279	2308	406	-21
Chronic liver disease	-1136	2282	2311	263	-18
Hypoglycaemia	-1139	2288	2317	148	-12
Nervous system disease	-1140	2289	2318	486	-11
Nephropathy	-1143	2296	2325	53	-4
Heart disease	-1143	2296	2325	1871	-4
Diabetic ketoacidosis	-1143	2297	2326	42	-3
Peripheral arterial disease	-1144	2299	2328	131	-1
Nothing (just baseline)	-1149	2306	2329	0	0
Neuropathy	-1145	2300	2329	153	0
Diabetic retinopathy	-1146	2302	2332	225	3
Immunodeficiency	-1148	2305	2334	28	5
Stroke	-1148	2306	2335	204	6
Foot ulcer	-1149	2308	2337	59	8

AIC = Akaike Information Criterion, BIC = Bayesian Information Criterion

As may be expected given the pathology of COVID-19, respiratory system disease was the comorbidity most strongly associated with the primary outcome (Table 1), followed by microvascular disease. Chronic kidney and liver disease also had strong associations. DR was not strongly associated with the outcome and using BIC, the association was lower than the baseline without it.

Thirdly, we asked how this comorbidity ranking was affected by the inclusion of hospital admissions as proxy for general health.

### Hospital admissions

Table 3 shows model fit (log likelihood): static comparison of models per comorbidity adjusted for full extended baseline, including admissions (log transformed).

**Table 3:** Static comparisons, extended baseline (age, sex, diabetes duration, ethnicity, deprivation, BMI, smoking, systolic blood pressure, hospital admissions (log), sorted by BIC with best performing models at the top

	Log-Likelihood	AIC	BIC	*n* (comorbidity)	BIC delta over baseline
Respiratory system disease	-1099	2227	2308	1011	-5
Microvascular disease	-1101	2231	2312	619	-1
Chronic liver disease	-1101	2230	2312	263	-1
Nothing (just baseline)	-1106	2238	2313	0	0
Chronic kidney disease	-1102	2232	2313	406	0
Diabetic ketoacidosis	-1102	2232	2313	42	0
Hypoglycaemia	-1103	2233	2315	148	2
Nephropathy	-1104	2235	2317	53	4
Nervous system disease	-1104	2236	2317	486	4
Peripheral arterial disease	-1105	2237	2319	131	6
Diabetic retinopathy	-1105	2239	2320	225	7
Neuropathy	-1106	2239	2320	153	7
Immunodeficiency	-1105	2239	2320	28	7
Foot ulcer	-1106	2239	2321	59	8
Stroke	-1106	2240	2321	204	8
Heart disease	-1106	2240	2321	1871	8

AIC = Akaike Information Criterion, BIC = Bayesian Information Criterion

With hospital admissions in the extended baseline, comorbidities do not add much information to the model (Table 3). As before, respiratory disease is most strongly associated with the outcome with chronic liver disease and microvascular disease second. Comparable analysis was undertaken excluding hospital admissions to examine the influence of other elements of the extended baseline with similar results (SM additional analyses).

Finally we asked which group of comorbidities was most informative about severe outcomes of COVID-19.

### Most informative comorbidities

**Fig. 2:**
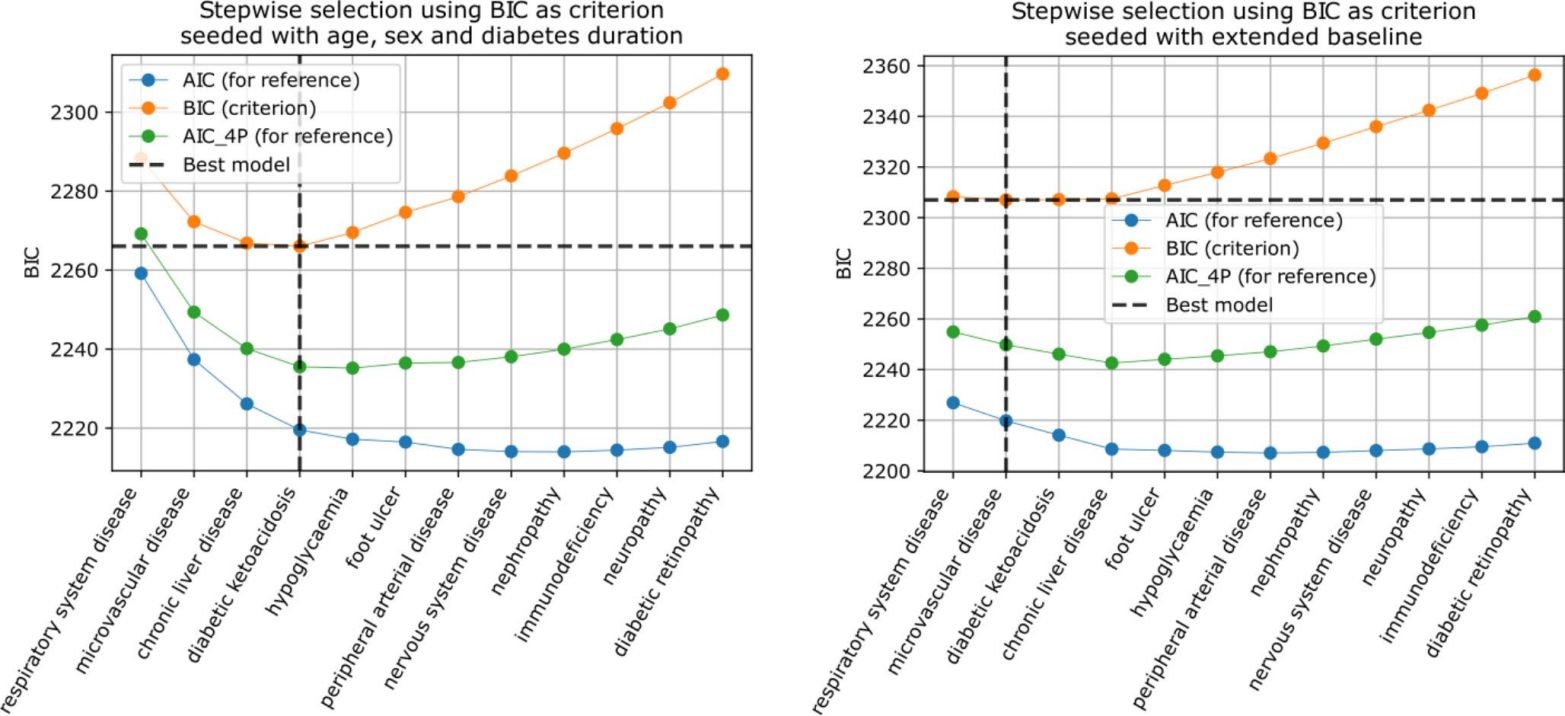
Stepwise selection result, seeded with (L) simple baseline (age, sex, diabetes duration) and (R) extended baseline (age, sex, diabetes duration, ethnicity, deprivation, BMI, smoking, systolic blood pressure, hospital admissions). Note: only comorbidities were considered in this selection

Using the simple baseline (age, sex and diabetes duration), selection favoured respiratory disease, followed by microvascular disease, chronic liver disease and diabetic ketoacidosis (Fig. 2 left). It was notable that, in contrast to the static comparisons reported above, chronic kidney disease was not selected, indicating that if we already knew the patient had respiratory, microvascular and liver disease, then it did not add much additional information.

Using the extended baseline, again we observed respiratory disease ranked highest followed by microvascular disease (Fig. 2 right). Using this baseline with log hospital admissions, the BIC barely improved, although microvascular disease was indeed selected.

In summary, respiratory and microvascular disease were consistently the most informative comorbidities for outcome prediction. Further, and importantly, microvascular disease was informative even if we already had respiratory disease in our model, which was not revealed in the static comparisons for research questions two and three.

### Beyond binary comorbidity encoding

Thus far, we used a binary indicator for each of the comorbidities. Binary encoding is simple and widely used. However, it fails to capture how recently and severely a comorbidity affected a given person. Our initial analysis suggested that count of comorbidity codes or the time to the most recent code might be informative of severe outcomes.

To investigate this, we repeated the forward stepwise variable selection with three alternatives to the binary comorbidity coding. First, comorbidity code count and time to most recent code. Additionally, a transformed version of time encoding where the value smoothly decays from 1 (for a code the day before the diagnosis) to a minimum value of 0·25 (for a code that happened over 10 years ago). More details in SM.

We seeded the variable selection with baseline covariates (age, sex, diabetes duration). For robustness, we ran the selection using AIC as criterion and again using BIC, which favours more parsimonious models. Interestingly, both count and simple time encodings yielded a poorer model than the binary one. However, the weighted time encoding improved model fit over the binary encoding in terms of BIC by 8 when selecting for BIC and AIC by 11 when selecting for AIC. The binary and weighted time encoding models selected the same number of variables, so the log likelihood of the weighted time encoding model was improved by 4 and 5·5 respectively. Interestingly, both the binary and weighted time encodings led to respiratory system disease and microvascular disease being selected first, whereas the other two, poorer performing encodings select other comorbidities first.

## Discussion

It was established early in the COVID-19 pandemic that people with diabetes were at additional risk of severe outcomes. This study further tests the theories proposed in early clinical studies around the association between DR, microvascular disease and severe outcomes on a large cohort with extensive, linked, longitudinal data. We have shown an association between both DR alone and a broader collection of microvascular diseases and fatal or CCU-treated COVID-19. However, the strength of the associations was strongly influenced by other aspects of the individual’s health which were difficult to differentiate in a large cohort based on routinely collected data. Our attempts to account for these through identification of relevant comorbidity groups and a proxy for general health through hospital admissions, greatly reduce the predictive power of DR in particular, and microvascular disease overall, although the latter was still selected after respiratory disease.

It is possible that some cases of microvascular (or other) disease developed coincidentally with severe COVID-19, complicating the analysis. Setting a stricter cut-off to identify prevalent comorbidities which clearly pre-existed COVID-19 infection in the individual may elucidate this point. However, using routinely collected data, available only when the individual interacts with healthcare systems, does mean that lengthy gaps in information on individuals occur. This was particularly problematic in the early days of the pandemic when people were under multiple lockdowns and advised to stay away from health services unless absolutely necessary.

This study focussed on the first year of the COVID-19 pandemic in the UK, before the vaccination programme was widely rolled out. However, even in this early period, the processes through which these data were generated went through several significant changes. Initially the UK conducted mainly confirmatory testing of symptomatic individuals, by definition excluding patients that were otherwise in good health. By summer 2020, this had shifted towards mass testing when more of this group would be detected and reducing this early selection bias among the “COVID-19 positive” group. In an attempt to include all COVID-19 cases, we have adopted a definition which added cases who never received a positive COVID-19 lab test but were coded as having COVID-19 through a hospital or death record. This represents a potential selection bias in the analysis.

Understanding, and therefore treatment, of severe COVID-19 improved significantly throughout the pandemic, but the sheer number of cases at certain points were reported to force some clinicians to make decisions based on service and equipment capacity rather than the clinical need of the individual [19]. The extent to which this affected outcomes in this cohort is impossible to determine from the available information, but incorporating date of infection and age or level of comorbidity at that point may reveal patterns which could explain this further.

Despite limitations in using large, cohort studies such as UKB (self-selecting participants, missing data, time delays between collection of some information and clinical outcomes), these data tell a familiar story of how COVID-19 disproportionately affected many specific sub-groups of populations around the world. Older people, men, people from some black and minority ethnic groups, people living in deprived areas were all at greater risk of being infected with COVID-19 and, in some cases, at higher risk of suffering severe outcomes of COVID-19. An illustration of the complex relationship between ethnicity and COVID-19 [20].

Early clinical studies looking at DR and COVID-19 outcomes were heterogeneous regarding the comorbidities they evaluated [10] but correspondence stressing the importance of looking beyond DR to microvascular disease more generally, followed [9]. Our findings add further weight to this line of investigation.

### Limitations

Our approach to identifying comorbidities using hospital records and their ICD10 codes was comprehensive (available across three UK countries with long term historical data for each participant). However, it is also somewhat blunt, and does not include all conditions which may affect patients’ prognosis due to severe COVID. Identifying diagnoses through these codes provides little information about the severity of disease or its clinical origin, for example the micro- or macrovascular origin of diagnosed diabetic foot ulcer. Our efforts to weight diagnostic codes by recency, did not reveal a stronger relationship between DR and severe COVID-19 outcomes.

The UKB cohort includes an extremely useful and rich set of demographic data however these were captured on recruitment to the study (with some follow up in subsequent years), which means that for most participants, values such as BMI or smoking status may be a decade or two out of date when they experienced COVID-19 or the primary outcome.

### Further work

This analysis could be conducted in other cohorts with access to other clinical data to examine the replicability of the findings, and extend the retinal phenotype descriptors to include rate of retinopathy change, or the impact of anti-diabetic treatments, disease severity or clinical origin on prognosis. Such analyses may benefit from being conducted on datasets with more patients with diabetes, so that sub-group analyses yield clinically and statistically meaningful results. The significant increase in rate of type I diabetes observed in young people during the pandemic suggested a possible diabetogenic mechanism in COVID-19 [21]. Further investigation within this age group may reveal useful information about the relationship between the conditions, and the physiological processes which ultimately lead to microvascular disease.

Our analysis could also be extended further within UKB. The proxy we included for general health (number of hospital admissions) could be refined and quantification of comorbidities could be added by looking at rate and overlap of codes to focus on more recent periods leading up to COVID-19 diagnosis. Vaccination status and antibody test results for the individual could be incorporated as covariates likely to influence the risk of severe outcomes, and more data from primary care may improve the analyses’ sensitivity. Other outcomes of interest could be tested including long COVID, which may be increasingly relevant as severe outcomes reduced in frequency following vaccine roll out and improved clinical management of the disease.

With global efforts continuing to vaccinate against COVID-19 infections and increasing rates of successful treatment, the disease may become endemic. Understanding how to rapidly identify people at greatest clinical risk remains a challenge.

## Supplementary Information

Below is the link to the electronic supplementary material.

Supplementary Material 1

Supplementary Material 2
