# Correction: microvascular disease and severe COVID-19 outcomes in UKBiobank participants with diabetes

**DOI:** 10.1007/s00592-025-02456-9

**Published:** 2025-02-03

**Authors:** Claire Tochel, Justin Engelmann, Ylenia Giarratano, Baljean Dhillon, Roly Megaw, Miguel O. Bernabeu

**Affiliations:** 1 Centre for Medical Informatics, Usher Institute, University of Edinburgh, Edinburgh, EH16 4UX UK; 2 Centre for Clinical Brain Sciences, Chancellor’s Building, University of Edinburgh, Edinburgh, EH16 4SB UK; 3 MRC Human Genetics Unit, University of Edinburgh, Edinburgh, EH4 2XU UK; 4 Princess Alexandra Eye Pavilion, NHS Lothian, Chalmers St, Edinburgh, EH3 9HA UK; 5 The Bayes Centre, University of Edinburgh, Edinburgh, EH8 9BT UK

**Correction: Acta Diabetologica** 10.1007/s00592-024-02420-z

In the original version of this article, the author’s name “Miguel O Bernabeu” was incorrectly written as “Miguel Bernabeu”.

And the Funding information section was incorrectly given as ‘This work was supported by a grant from Diabetes UK (20/0006221). The funders did not influence the study design or methodology’ and should have read ‘This work was supported by a grant from Diabetes UK (20/0006221). MOB also gratefully acknowledges funding from: Fondation Leducq Transatlantic Network of Excellence (17 CVD 03); EPSRC grant no. EP/X025705/1; British Heart Foundation and The Alan Turing Institute Cardiovascular Data Science Award (C-10180357); Fight for Sight (5137/5138); the SCONe projects funded by Chief Scientist Office, Edinburgh & Lothians Health Foundation, Sight Scotland, the Royal College of Surgeons of Edinburgh, the RS Macdonald Charitable Trust, and Fight For Sight. JE was supported by the United Kingdom Research and Innovation (grant EP/S02431X/1), UKRI Centre for Doctoral Training in Biomedical AI at the University of Edinburgh, School of Informatics.

The funders did not influence the study design or methodology’.

The original article has been corrected.

