# Supplementary material for: Microvascular disease and severe COVID-19 outcomes in UKBiobank participants with diabetes
Source: Acta Diabetol. 2024 Nov 21;62(3):293–301. doi: 10.1007/s00592-024-02420-z (PMC11872747; doi:10.1007/s00592-024-02420-z)
Supplement: Supplementary file 1 — Supplementary Material 1 [file 592_2024_2420_MOESM1_ESM.docx]

Microvascular disease and severe COVID-19 outcomes in UKBiobank participants with diabetes

Supplementary material

Contents

[Demographic details for study cohort 1](#_Toc175650157)

[Relationship between DR & microvascular disease and codes 2](#_Toc175650158)

[Accounting for code recency 3](#_Toc175650159)

[Modelling relationships 3](#_Toc175650160)

[Missing data 4](#_Toc175650161)

[Additional Analyses 4](#_Toc175650162)

### Demographic details for study cohort

**Table 4** Descriptive statistics for UKBiobank participants with diabetes according to whether they had COVID-19 and the primary outcome

|  | **No +ve COVID-19 test** | **1 or more +ve COVID-19 test** | |
| --- | --- | --- | --- |
|  |  | **Without 1y outcome** | **1y outcome** |
| **Total** | 47,353 (95%) | 1,989 (4%) | 489 (1%) |
| **Sex** |  |  |  |
| Female | 20,768 (44%) | 831 (42%) | 154 (31%) |
| Male | 26,585 (56%) | 1,158 (58%) | 335 (69%) |
| **Age, years** (sd) | 71·4 (7·4) | 68·8 (8·2) | 73·7 (7) |
| **BMI, kg/m^2^** (sd) | 30·9 (5·7) | 31·7 (5·8) | 32·1 (6·1) |
| **Ethnic group** |  |  |  |
| asian | 2,790 (6%) | 224 (11%) | 36 (7%) |
| black | 1,535 (3%) | 102 (5%) | 23 (5%) |
| mixed/other | 1,127 (2%) | 68 (3%) | 8 (2%) |
| unknown | 414 (1%) | 18 (1%) | 11 (2%) |
| white | 41,487 (88%) | 1,577 (79%) | 411 (84%) |
| **Deprivation quintile** |  |  |  |
| 1 (lowest) | 17,827 (38%) | 587 (30%) | 138 (28%) |
| 2 | 9,983 (21%) | 374 (19%) | 81 (17%) |
| 3 | 7,755 (16%) | 383 (19%) | 89 (18%) |
| 4 | 7,572 (16%) | 415 (21%) | 110 (22%) |
| 5 (highest) | 4,146 (9%) | 226 (11%) | 71 (15%) |
| unknown | 70 (0%) | 4 (0%) | NA (0%) |
| **Smoking status** |  |  |  |
| ever smoked | 24,124 (51%) | 1,010 (51%) | 305 (62%) |
| never smoked | 22,749 (48%) | 958 (48%) | 175 (36%) |
| unknown | 480 (1%) | 21 (1%) | 9 (2%) |
| **Diabetes duration, years** (sd) | 13·7 (10·6) | 13·5 (10·5) | 13·9 (11·4) |

Abbreviations: 1y= primary, BMI = Body Mass Index, sd = standard deviation

### Relationship between DR & microvascular disease and codes


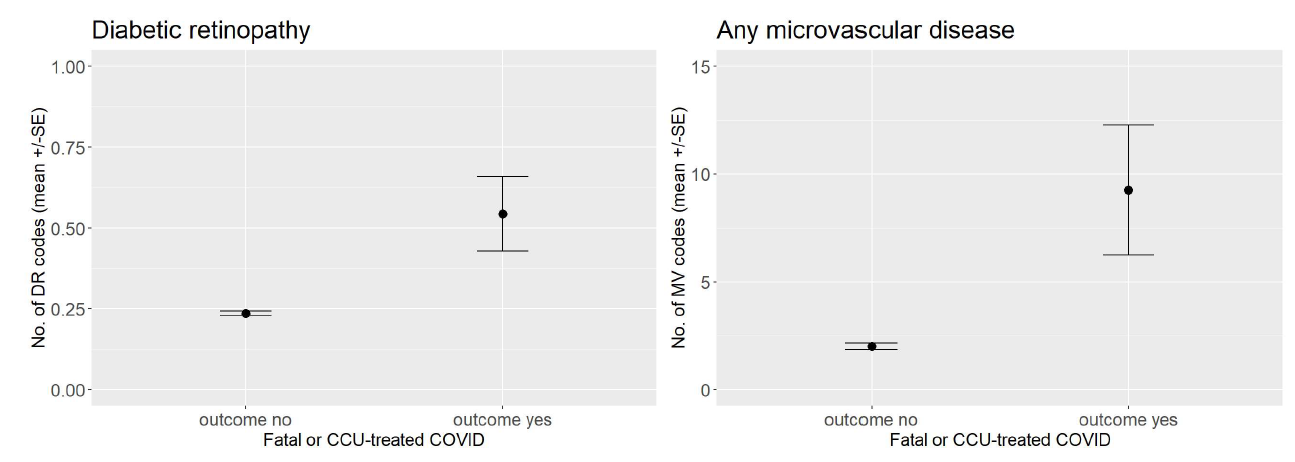


**Figure 3** No of diagnostic codes for DR (left) or any microvascular disease (right) by study outcome


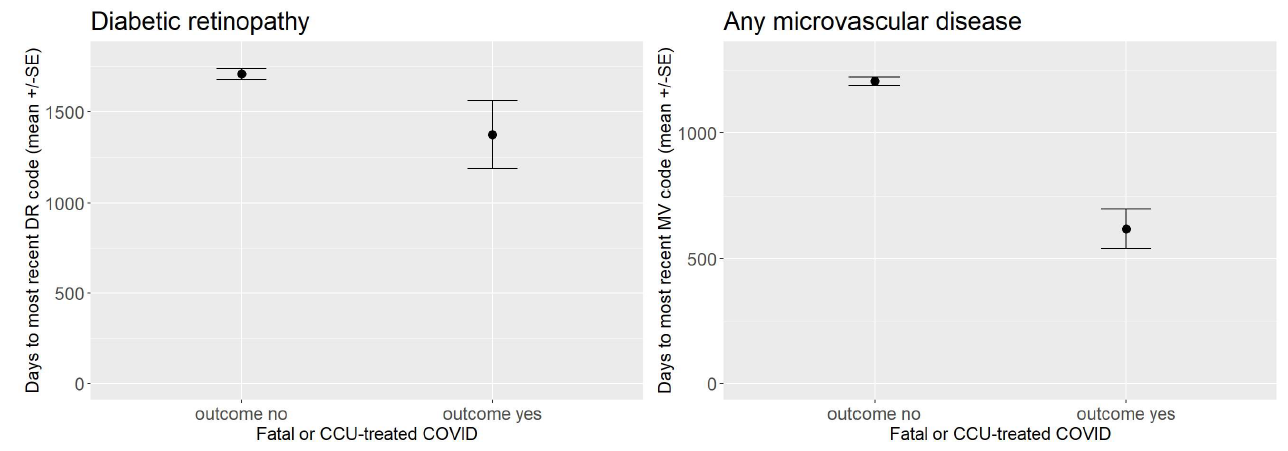


**Figure 4** Days to most recent diagnostic code for DR (left) or any microvascular disease (right) by study outcome

### Accounting for code recency

Transformed time to most recent code: value is given by $f\left( t \right)=\frac{1}{2}\left( 1+\cos\left( \pi\times\frac{t}{10} \right) \right)\times\left( 1-0\cdot25 \right)+0\cdot25$ where t is the time since the given code in years (Figure 5).


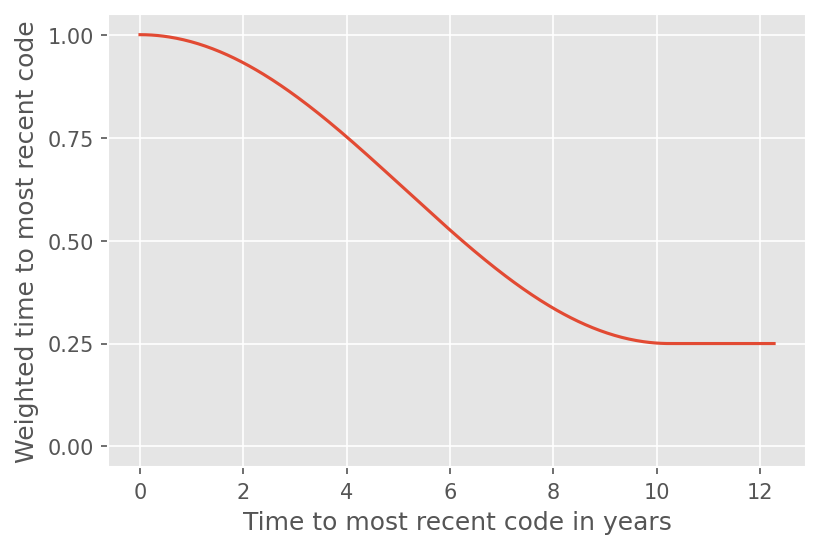


**Figure 5** Weighted time to most recent code

### Modelling relationships

The commonly used AIC expresses that each additional parameter should improve the log likelihood by at least 1. The relative likelihood of the two models is given by $\exp\left( \frac{AIC_{Binary}-AIC_{Weighted}}{2} \right)$. Thus, the model with weighted comorbidity coding is $\exp\left( \frac{8}{2} \right)\approx54\cdot6$ times more likely when optimising for BIC and $\exp\left( \frac{11}{2} \right)\approx244\cdot7$ more likely when optimising for AIC. Thus, the observed improvements can be considered meaningful.

**Table 5** Results of stepwise selection with different comorbidity encodings using BIC as criterion

|  | **No of variables selected** | **Selected variables** | **Best BIC** | **Best AIC** |
| --- | --- | --- | --- | --- |
| **Weighted time** | 4 | respiratory system disease (weighted), microvascular disease (weighted), diabetic ketoacidosis (weighted), chronic liver disease (weighted) | 2258·0 | 2211·0 |
| **Binary** | 4 | respiratory system disease, microvascular disease, chronic liver disease, diabetic ketoacidosis | 2266·0 | 2220·0 |
| **Count** | 4 | heart disease (weighted), chronic liver disease (weighted), immunodeficiency (weighted), hypoglycaemia (weighted) | 2290·0 | 2243·0 |
| **Time to most recent** | 3 | nervous system disease (weighted), heart disease (weighted), chronic liver disease (weighted) | 2309·0 | 2268·0 |

Abbreviations: AIC = Akaike Information Criterion, BIC = Bayesian Information Criterion

**Table 6** Results of stepwise selection with different comorbidity encodings using AIC as criterion

|  | **No. of variables selected** | **Selected variables** | **Best BIC** | **Best AIC** |
| --- | --- | --- | --- | --- |
| **Weighted time** | 9 | respiratory system disease (weighted), microvascular disease (weighted), diabetic ketoacidosis (weighted), chronic liver disease (weighted), foot ulcer (weighted), hypoglycaemia (weighted), peripheral arterial disease (weighted), immunodeficiency (weighted), nervous system disease (weighted) | 2279·0 | 2203·0 |
| **Binary** | 9 | respiratory system disease, microvascular disease, chronic liver disease, diabetic ketoacidosis, hypoglycaemia, foot ulcer, peripheral arterial disease, nervous system disease, nephropathy | 2290·0 | 2214·0 |
| **Count** | 8 | heart disease (weighted), chronic liver disease (weighted), immunodeficiency (weighted), hypoglycaemia (weighted), nervous system disease (weighted), respiratory system disease (weighted), diabetic ketoacidosis (weighted), neuropathy (weighted) | 2306·0 | 2236·0 |
| **Time to most recent** | 5 | nervous system disease (weighted), heart disease (weighted), chronic liver disease (weighted), nephropathy (weighted), foot ulcer (weighted) | 2316·0 | 2264·0 |

Abbreviations: AIC = Akaike Information Criterion, BIC = Bayesian Information Criterion

###

### Missing data

**Table 7** Missing rate and imputed values

|  | **missing** | **median** | **mode** |
| --- | --- | --- | --- |
| **sex** | 0% | ·· | ·· |
| **age** | 0% | ·· | ·· |
| **admSince98** | 0% | ·· | ·· |
| **diab_dur** | 0% | ·· | ·· |
| **eth1stAss** | 0·36% | ·· | British |
| **ethRecode** | 0·36% | ·· | white |
| **smoke1stAss** | 0·36% | ·· | Never |
| **townsendDep** | 0·15% | -1·35 | ·· |
| **townsendQ** | 0·15% | ·· | 1 (lowest) |
| **bmi1stAss** | 1·07% | 27·59 | ·· |
| **bmiCat** | 1·07% | ·· | overweight |
| **bpSys** | 0·52% | 136·0 | ·· |
| **bpCat** | 0·52% | ·· | normal BP |

### Additional Analyses

Akaike Information Criterion (AIC) and Bayesian Information Criterion (BIC)

- AIC = 2p - 2 log likelihood
- BIC = p * ln(n) - 2 log likelihood

where p is the number of parameters that we fit, and n is the number of samples. So both AIC/BIC are measures of model fit adjusted for the number of parameters. BIC is typically much more conservative.

McGurnaghan *et al* use a "stringent" non-standard version of the AIC with a penalty of 4p, i.e. twice as high as standard AIC. ^4^ From their appendix:


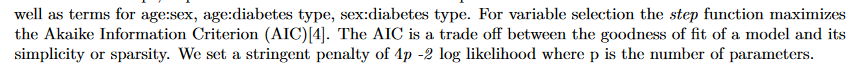


The documentation they reference with [4] notes that only 2p gives "genuine AIC".

For simple baseline, we select log(adm) first, which is what we would expect. However, both respiratory and microvascular disease still make the cut.

For extended baseline, we only select respiratory and microvascular disease but no other columns – which also makes sense, as the most informative columns are already in the extended baseline.


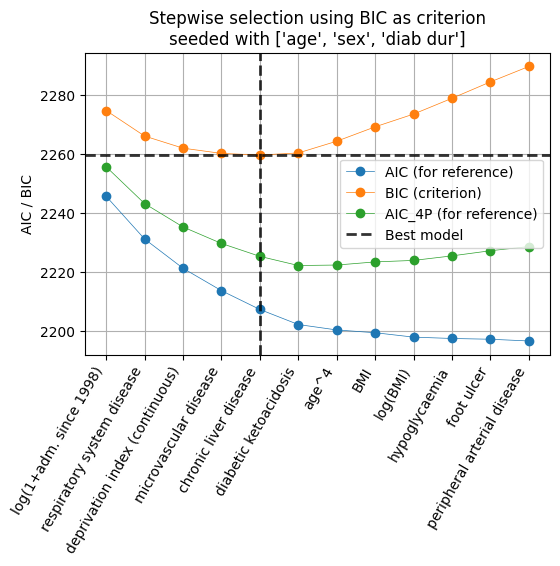

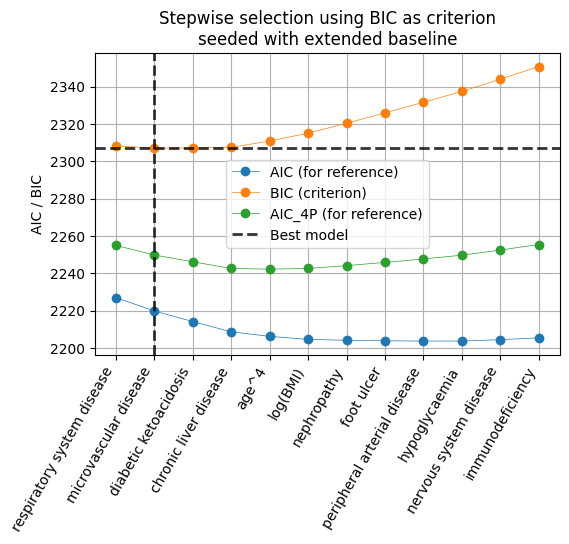


**Figure 6** Stepwise selection considering all columns (rather than just comorbidities, simple (left) and extended (right) baseline

**Table 8** Static comparisons – simple baseline with number of admissions (log)

|  | **Log-Likelihood** | **AIC** | **BIC** | **n (comorbidity)** | **BIC delta over baseline** |
| --- | --- | --- | --- | --- | --- |
| **respiratory system disease** | -1110 | 2231 | 2266 | 1011 | -9 |
| **microvascular disease** | -1113 | 2237 | 2272 | 619 | -3 |
| **chronic liver disease** | -1112 | 2237 | 2272 | 263 | -3 |
| **chronic kidney disease** | -1113 | 2239 | 2274 | 406 | -1 |
| **Nothing (just baseline)** | -1118 | 2246 | 2275 | 0 | 0 |
| **diabetic ketoacidosis** | -1114 | 2240 | 2275 | 42 | 0 |
| **hypoglycaemia** | -1114 | 2241 | 2276 | 148 | 1 |
| **nephropathy** | -1115 | 2243 | 2278 | 53 | 3 |
| **nervous system disease** | -1116 | 2244 | 2279 | 486 | 4 |
| **peripheral arterial disease** | -1116 | 2245 | 2280 | 131 | 5 |
| **diabetic retinopathy** | -1117 | 2247 | 2281 | 225 | 6 |
| **neuropathy** | -1117 | 2247 | 2282 | 153 | 7 |
| **foot ulcer** | -1118 | 2248 | 2282 | 59 | 7 |
| **immunodeficiency** | -1117 | 2247 | 2282 | 28 | 7 |
| **stroke** | -1118 | 2248 | 2283 | 204 | 8 |
| **heart disease** | -1118 | 2248 | 2283 | 1871 | 8 |

**Table 9** Static comparisons - extended baseline without number of admissions (log)

|  | **Log-Likelihood** | **AIC** | **BIC** | **n (comorbidity)** | **BIC delta over baseline** |
| --- | --- | --- | --- | --- | --- |
| **respiratory system disease** | -1112 | 2251 | 2326 | 1011 | -29 |
| **microvascular disease** | -1117 | 2259 | 2335 | 619 | -20 |
| **chronic kidney disease** | -1119 | 2264 | 2340 | 406 | -15 |
| **chronic liver disease** | -1120 | 2267 | 2342 | 263 | -13 |
| **hypoglycaemia** | -1122 | 2270 | 2346 | 148 | -9 |
| **nervous system disease** | -1122 | 2270 | 2346 | 486 | -9 |
| **nephropathy** | -1125 | 2277 | 2352 | 53 | -3 |
| **diabetic ketoacidosis** | -1125 | 2276 | 2352 | 42 | -3 |
| **Nothing (just baseline)** | -1131 | 2285 | 2355 | 0 | 0 |
| **peripheral arterial disease** | -1127 | 2280 | 2356 | 131 | 1 |
| **heart disease** | -1127 | 2281 | 2356 | 1871 | 1 |
| **neuropathy** | -1128 | 2281 | 2357 | 153 | 2 |
| **diabetic retinopathy** | -1129 | 2283 | 2359 | 225 | 4 |
| **stroke** | -1130 | 2286 | 2361 | 204 | 6 |
| **immunodeficiency** | -1130 | 2285 | 2361 | 28 | 6 |
| **foot ulcer** | -1131 | 2287 | 2363 | 59 | 8 |


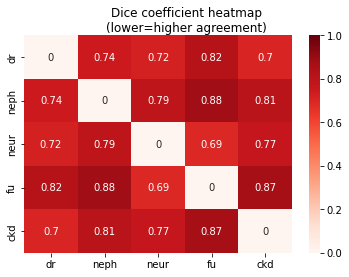


**Figure 7** Dice coefficient heatmap of different comorbidities
