# Supplementary material for: Microvascular disease and severe COVID-19 outcomes in UKBiobank participants with diabetes
Source: Acta Diabetol. 2024 Nov 21;62(3):293–301. doi: 10.1007/s00592-024-02420-z (PMC11872747; doi:10.1007/s00592-024-02420-z)
Supplement: Supplementary file 2 — Supplementary Material 2 [file 592_2024_2420_MOESM2_ESM.docx]

Conflicts of interest/Competing interests

The authors have no relevant financial or non-financial interests to disclose.
